# The Association of *IFI27* Expression and Fatigue Intensification during Localized Radiation Therapy: Implication of a Para-Inflammatory Bystander Response

**DOI:** 10.3390/ijms140816943

**Published:** 2013-08-16

**Authors:** Chao-Pin Hsiao, Maria Araneta, Xiao Min Wang, Leorey N. Saligan

**Affiliations:** 1 Frances Payne Bolton School of Nursing, Case Western Reserve University, 2120 Cornell Road, Cleveland, OH 44106, USA; E-Mail: hsiaochaopin@gmail.com; 2 National Institute of Nursing Research, National Institutes of Health, 9000 Rockville Pike, Building 10, Room 2-1339, Bethesda, MD 20892, USA; E-Mails: maria.araneta@nih.gov (M.A.), xmwang@mail.nih.gov (X.M.W.)

**Keywords:** fatigue, gene expression, interferon, prostate cancer, radiation

## Abstract

The mechanisms behind fatigue intensification during cancer therapy remain elusive. The interferon alpha-inducible protein 27 (*IFI27*) was the most up-regulated gene based on our previous microarray data in fatigued men with non-metastatic prostate cancer receiving localized external beam radiation therapy (EBRT). The purpose of this study was to confirm the *IFI27* up-regulation and determine its association with fatigue intensification during EBRT. Peripheral blood samples and fatigue scores were collected at three time points—prior to EBRT, at midpoint, and at completion of EBRT. Confirmatory quantitative real time polymerase chain reaction (qPCR) and enzyme-linked immunosorbent assay (ELISA) were utilized to verify the microarray results. Subjects were a total of 40 Caucasian men with prostate cancer; 20 scheduled for EBRT (65.6 ± 7.5 years old), and 20 on active surveillance as controls (62.8 ± 6.1 years old). Significant *IFI27* expression overtime during EBRT was confirmed by qPCR (*p* < 0.5), which correlated with fatigue scores during EBRT (*R* = −0.90, *p* = 0.006). Alterations in mechanisms associated with immune response and mitochondrial function that explain the up-regulation of *IFI27* may provide an understanding of the pathways related to the intensification of fatigue during localized radiation therapy.

## 1. Introduction

Improvement in prostate cancer treatment, especially with the use of modulated radiation therapy has increased cure and survival rates [1]. However, side effects associated with these treatments, such as fatigue are commonly reported [2,3]. Fatigue severity in most men with non-metastatic prostate cancer is known to increase significantly during the course of radiation therapy (RT) peaking at midpoint and declining after completion of the RT [4]. The etiology behind the development and intensification of fatigue while receiving cancer treatment remains unknown. A recent review revealed the lack of longitudinal studies that can explain physiologic mechanisms behind intensification of fatigue during cancer therapy [5]. Although studies reported associations between immune and inflammatory markers with the worsening intensity of cancer-related fatigue (CRF) during therapy [6–10], no compelling data between a specific biomarker and CRF has been established, contributing to its inadequate clinical management.

The peripheral blood has been used to investigate the etiology of CRF; similarly peripheral blood serves as the best medium of bystander response during radiation therapy [11]. In radiation therapy, release of reactive oxygen species and reactive nitrogen species during irradiation induces detrimental cellular damage, not only to irradiated cells but also to non-targeted cells that receive signals from irradiated cells, causing short and long term bystander effects through cytokine stimulation to respond to tissue damage [12]. There is a growing area of research asserting that cancer- and cancer-treatment related fatigue is driven by pro-inflammatory cytokines (e.g., IL-6, IL-1β, TNF-α) and inflammatory pathways (e.g., CRP, IL-1RA) as observed using animal [13,14] and clinical models [6,15]. A recent review characterized the inflammatory response from bystander cells as a para-inflammatory response to low dose radiation [16]. Para-inflammatory response can become chronic if tissue malfunction is persistent [17], and it may contribute to cancer-treatment related sickness behavior [18].

We recently reported profiles of gene expression changes over time during radiation therapy in men treated with non-metastatic prostate cancer, suggesting that mitochondrial dysfunction [19] and neuroinflammatory pathways [20] may play a role in fatigue development and intensification during external beam radiation therapy (EBRT). In our previous gene expression study, the interferon alpha-inducible protein 27 (*IFI27*) is the most up-regulated gene whole blood RNA of men with non-metastatic prostate cancer receiving localized EBRT [20]. The purpose of this study was to confirm the *IFI27* up-regulation and determine its association with fatigue intensification during EBRT, as well as to learn possible pathways, based on the known physiologic functions of *IFI27* that can provide clues of the role of bystander response to fatigue intensification during EBRT.

## 2. Results

The total sample of 40 men with non-metastatic prostate cancer consisted of 20 men who received EBRT and 20 age-, gender-, race- matched controls, who were on active surveillance for their prostate cancer. Table 1 describes the demographic and clinical characteristics of the study participants. The mean age of EBRT subjects was 65.6 years (±7.5), which was within ±5 years from the matched controls (62.8 ± 6.1). In the EBRT group, 17 (85%) received androgen deprivation therapy two months before EBRT and 2 had a radical prostatectomy more than 6 months before scheduled to receive EBRT. None of the participants reached the cutoff score for depression using the Hamilton Depression Scale (HAM-D). About 90% (*n* = 18/20) of EBRT subjects received a total dose of 75.6 Gray of EBRT, while the rest received a total dose of 68.4 Gray. PSA was significantly higher (*p* < 0.02) in the study subjects compared to controls related to their higher risk of disease.

### 2.1. Fatigue during EBRT

**Figure 1:**
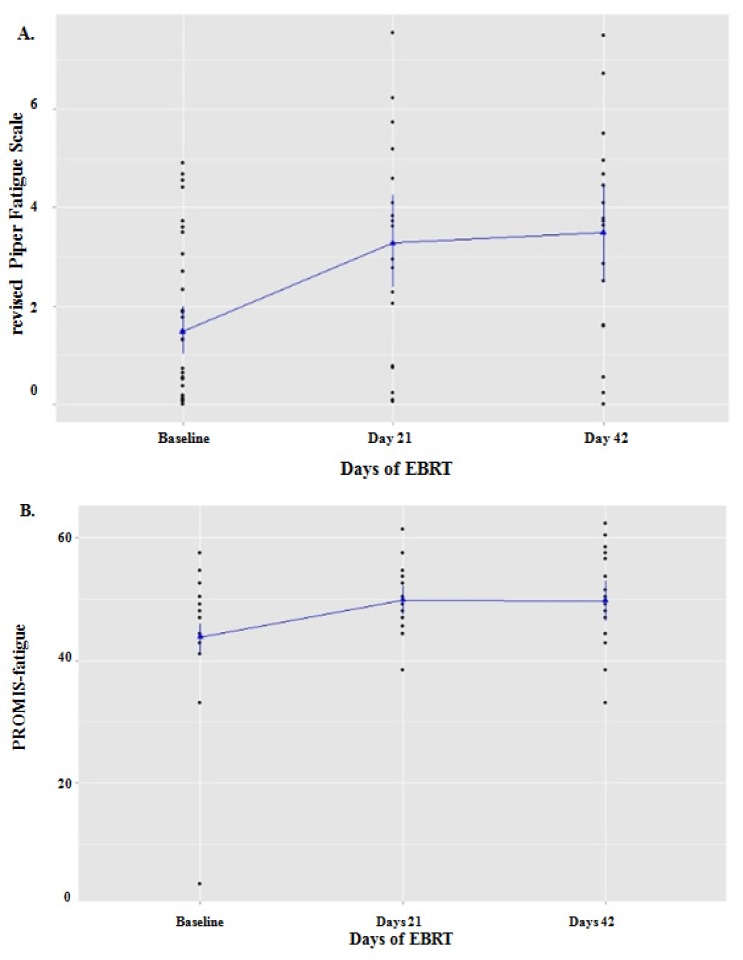
Fatigue scores of the sample. (**A**) Fatigue scores of 20 men with non-metastatic prostate cancer significantly changed (*p* = 0.001) from baseline to midpoint (Day 21), and completion (Day 42) of external beam radiation therapy (EBRT) as measured by the revised Piper Fatigue Scale (*rPFS*); (**B**) Similarly, fatigue scores of the same 20 subjects significantly changed (*p* = 0.002) from baseline to Day 21, and Day 42 of EBRT as measured by the Patient Reported Outcomes Measurement Information System-Fatigue subscale (PROMIS-F).

The *rPFS* fatigue scores of EBRT subjects (mean = 1.5 ± 1.6) and matched controls (mean = 1.46 ± 1.73, *p* = 0.93) were similar at baseline. Similarly, the mean PROMIS fatigue *T*-score at baseline for subjects (45.9 ± 6.3) and matched controls (41.7 ± 9.5) were not significantly different (*p* = 0.16). The mean fatigue score of EBRT participants increased significantly over time on both *rPFS* (*F* = 13.22, *p* < 0.001) and PROMIS-F (*F* = 7.27, *p* < 0.002) during EBRT. Compared to baseline (1.5 ± 1.6), the *rPFS* scores increased significantly at midpoint (3.27 ± 2.2, *p* < 0.001) and at completion of EBRT (3.49 ± 2.29, *p* = 0.001). The *rPFS* scores did not significantly change from midpoint to completion of EBRT (*p* = 0.93). Compared to baseline (45.85 ± 6.34), PROMIS-F scores increased significantly at midpoint (49.84 ± 5.47, *p* = 0.001) and at completion of EBRT (49.69 ± 7.59, *p* = 0.002). There was no significant difference in PROMIS-F scores from midpoint to completion of EBRT (*p* = 0.64). High variability in subjects’ fatigue scores was observed. A 3-point change in fatigue score has been found to be clinically important in a previous study [21]. The *rPFS* and PROMIS-F scores were highly correlated at each time point (*r* = 0.65–0.91, *p* < 0.01). Figure 1A,B illustrate changes in *rPFS* and PROMIS-F scores.

### 2.2. Gene Expression by Microarray

Four hundred sixty three probesets (178 up-regulated and 285 down-regulated) were differentially expressed over time after the probesets passed filtering criteria of 1% false discovery rate (FDR) and a slope of 0.07 or more (over 2.6-fold change, *p* < 0.0003), which we recently reported [20]. The interferon alpha-inducible protein 27 (*IFI27*) was the most up-regulated gene in the list (expression value = 0.774, *p* < 0.0001), and was selected for further confirmation based on its association with inflammation and mitochondrial dysfunction, both mechanisms were proposed to be physiologic mechanisms behind CRF [19,20]. The average log 10 expression of the *IFI27* probeset from that study showed a significant upward trend of *IFI27* expression during EBRT (*p* < 0.001). Table S1 shows the top 10 differentially expressed genes by microarray.

### 2.3. Confirmation of *IFI27* Expression during EBRT

**Figure 2:**
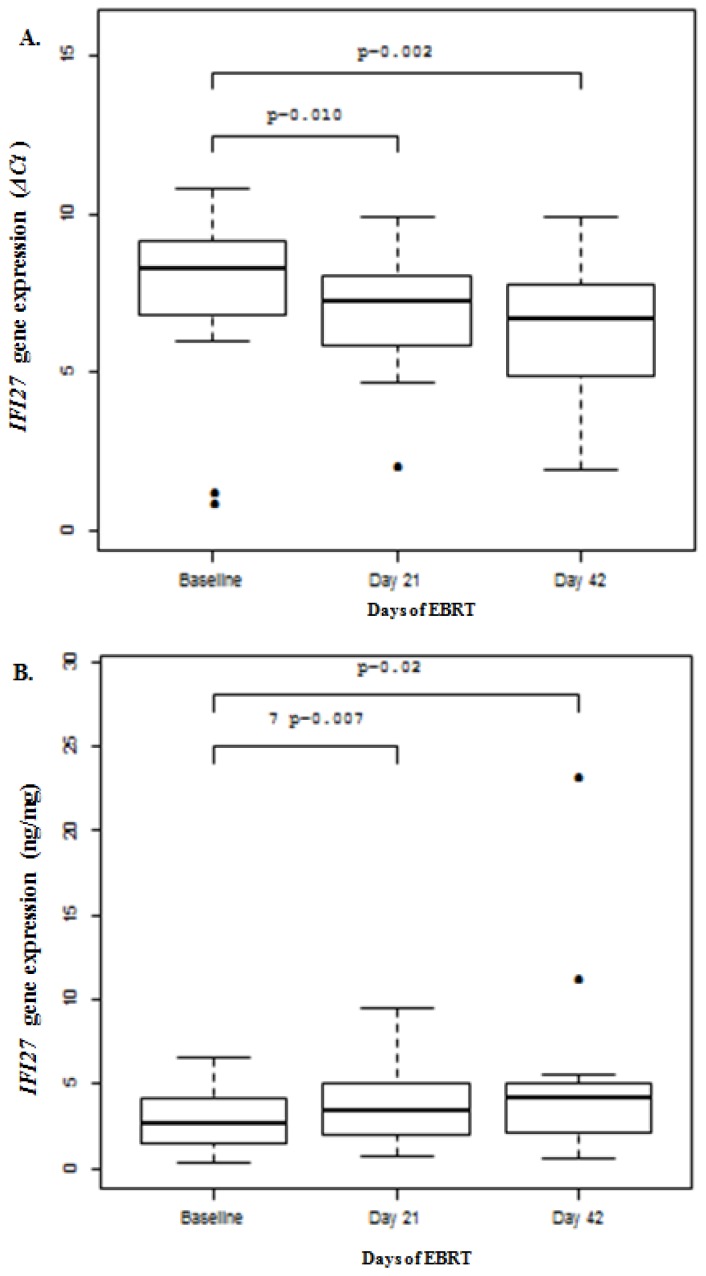
Confirmation of *IFI27* expression. (**A**) Interferon alpha-inducible protein 27 (*IFI27*) significantly changed from baseline to day 21 and day 42 of external beam radiation therapy (EBRT) using quantitative real time polymerase chain reaction (qPCR) as measured by delta cycle time (Δ*Ct*, an approximation method). *Ct* representes level of gene expression; the less *Ct* value, the higher the gene expression level. The *Ct* value of IFI27 decreases over time indicating an over expression of IFI27 over time; (**B**) There was also a significant change in the concentration (ng/mg) of the human interferon alpha-inducible protein 27, mitochondrial isoform 1 as measured by enzyme-linked immunosorbent assay (ELISA) from baseline to day 21, and day 42 of EBRT.

Further confirmation revealed no significant differences in *IFI27* gene (*p* = 0.56) and protein (*p* = 0.54) expressions between EBRT subjects and matched controls at baseline using qRT-PCR and ELISA, respectively. Significant up-regulation of *IFI27* during EBRT was confirmed (*F* = 9.55, *p* = 0.002) by qRT-PCR. *IFI27* gene expression increased significantly from baseline to D21 (*p* = 0.01) and to D42 of EBRT (*p* = 0.002, Figure 2A). Similarly, IFI27 protein expression increased from baseline to D21 (*p* = 0.007) and to D42 of EBRT (*p* = 0.02, Figure 2B), as measured by ELISA. Please refer to Figure 2.

### 2.4. Correlation between Fatigue and *IFI27* Expression

**Figure 3:**
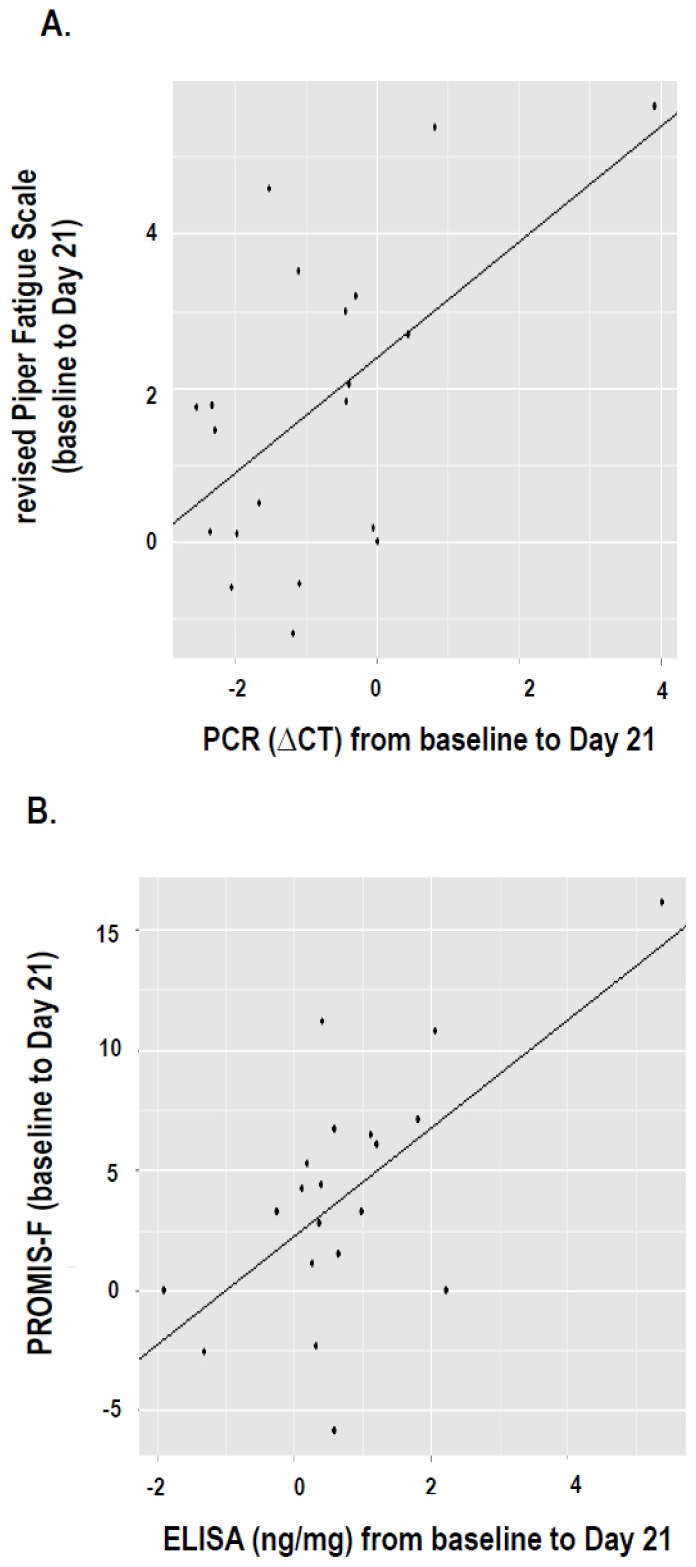
Correlations between changes in fatigue scores and gene/protein expressions of *IFI27*. (**A**) Changes in fatigue measured by the revised Piper Fatigue Scale was significantly associated (*R* = 0.56, *p* = 0.01) with changes in the expression of interferon alpha-inducible protein 27 (*IFI27*) using qPCR (from baseline to midpoint of external beam radiation therapy); (**B**) Changes in fatigue measured by the Patient Reported Outcomes Measurement Information System-Fatigue subscale (PROMIS-F) was significantly associated (*R* = 0.64, *p* = 0.01) with changes in ELISA interferon alpha-inducible protein 27 (*IFI27*) concentration (from baseline to midpoint of external beam radiation therapy).

A significant correlation between changes in *rPFS* fatigue scores and *IFI27*gene expression using qRT-PCR (*ΔCT*) was observed from baseline to D21 of EBRT (*R* = 0.56, *p* = 0.001). No other significant correlation was noted between the variables at any study time point. However, changes in IFI27 protein concentration was significantly correlated with changes in fatigue (*R* = 0.64, *p* = 0.001) as measured by the PROMIS-F from baseline to D21 of EBRT. Figures 3A, B illustrate the association between changes in *IFI27* gene/protein expressions and changes in fatigue score. Please refer to Figure 3.

## 3. Discussion

Using microarray technique as an unbiased, hypothesis-generating approach, this study gained new insights into the possible role of bystander response during EBRT. EBRT is a well-established treatment modality delivering radiation doses to tumor sites. A local RT-induced increase in type 1 IFNs (α and β) in the tumor microenvironment, produced by myeloid cells in an autocrine fashion, was recently noted to be an essential factor in tumor regression [22]. Peripheral blood serves as the medium for bystander effects of EBRT. Using whole blood RNA, we observed that the most up-regulated gene that was significantly associated with fatigue intensification during EBRT was *IFI27*. This gene is known to induce apoptosis by sending extracellular signals to activate other pro-apoptotic genes [23]. *IFI27* gene encodes a putative highly hydrophobic membrane protein [24], and is highly induced by interferon-alpha/beta (IFN-α/β) [25]; both cytokines alter immune response by acting as pleiotropic cytokines in generating long-lasting immune response through activities of T lymphocytes and dendritic cells [26]. This finding provides some empirical support that fatigue intensification during EBRT is a bystander response to radiation, and this bystander response can be explained by the upregulation of *IFI27*, which influences mitochondrial function and immune response, both mechanisms we believe to be involved in CRF.

It is believed that IFN-α, induced by *IFI27* [27], stimulates myeloid dendritic cells to activate IL-12, which in turn prompts T-cells to secrete IFN-γ, and arms NK cells and CD8+ cytotoxic T lymphocytes (CTL) with perforin A and granzyme B for killing tumor targets [28]. Fatigue has been associated with low levels of perforin in NK cells [29]. Decreased NK cell activity [30], decreased frequency of myeloid dendritic cells in circulating activated T lymphocytes [31], and higher level of CD4+ T cells that increased in response to stress were observed in fatigued breast cancer survivors [32]. The immune pathways that involve activities of *IFI27*-induced proteins can shed some light on the mechanisms behind fatigue intensification during localized radiation therapy.

IFN-α therapy has been approved by the Food and Drug Administration (FDA) for treating solid tumors such as malignant melanoma, Kaposi’s sarcoma related to acquired immune deficiency syndrome, and hematologic malignancies such as aggressive follicular non-Hodgkin’s lymphoma and chronic myelogenous leukemia [28]. It has been shown to prolong disease-free survival in melanoma patients [33]. However, IFN-α therapy is associated with multiple toxicities such as fatigue, anorexia, fever, nausea, and chills [34] and is often administered at a lower dose or as an adjuvant therapy to reduce these toxicities [28]. An association between fatigue and overexpression of 2′-5′-oligoadenylate synthetase 2 (OAS2), a gene linked to chronic fatigue syndrome, was noted in cancer patients receiving chronic IFNα therapy [35]. This finding and results of our study suggest that downstream pathways (e.g., IFNs-α and β, IL-12, NK cells, and CTL) involved in innate and adaptive immune responses that are activated by *IFI27* may provide some mechanism that influence the intensification of fatigue in this population.

The change of fatigue scores using *rPFS* was associated with the change of *IFI27* PCR expression, while the change of fatigue scores using PROMIS-F was associated with the change of *IFI27* protein expression. Although *rPFS* and PROMIS-F are highly correlated in this study, their associations to potential biological markers of fatigue differ; hence, further investigation is warranted. We have reported previous associations between *rPFS*, PROMIS-F, and molecular-genetics findings [19,20]. More longitudinal investigations are needed to confirm validity and reliability of these tools in assessing relationships of potential physiological markers with fatigue.

This study was conducted in a tertiary research setting with a semi-selective patient population; therefore, the results may not be generalizable. Another limitation to this study is the small sample size. Additionally, collecting data at one time point from the control group limited our ability to longitudinally compare the trajectory of fatigue symptoms and gene/protein expressions in prostate cancer not receiving any treatment from EBRT subjects.

Our hypothesis-generating finding confirms the association of *IFI27* upregulation and fatigue intensification during EBRT, but it does not prove causation. Further investigation is necessary to determine the direct link of mitochondrial dysfunction and para-inflammation from *IFI27* upregulation with fatigue intensification during cancer therapy. The change of fatigue symptoms we associated our gene expression data with, has been reported to be the minimum change in fatigue scores that is large enough to influenceclinical care [20]. Conducting an intervention targeting *IFI27* to reduce fatigue symptom during EBRT will confirm the clinical relevance of our findings. Understanding the etiology of fatigue during cancer therapy is critical because CRF persists even at survivorship [36]. About 71% of our participants with high fatigue during EBRT had persistent high fatigue symptoms one year post EBRT, which is consistent with previous findings [4].

## 4. Methods/Experimental Section

This study was approved by the Institutional Review Board of the National Institutes of Health (NIH), Bethesda, MD, USA (NCT00852111). Subjects were enrolled from May 2009 to September 2011 and data were collected at three time points: baseline (prior to EBRT, D0), midpoint (days 19–21, D21), and completion (days 38–42, D42) of EBRT. To distinguish that differential gene expression observed in this study is related to EBRT and not to stage of cancer, age-, gender-, race-matched patients on active surveillance for their non-metastatic prostate cancer were used as controls for the comparison of fatigue and gene/protein expressions at baseline (pre-EBRT). Patients and controls were excluded from the study if they had progressive disease causing significant fatigue; experienced psychiatric disease within five years; had uncorrected hypothyroidism or anemia; took sedatives, steroids, or non-steroidal anti-inflammatory agents; or had a second malignancy.

Self-report fatigue was measured at each time point using the validated revised Piper Fatigue Scale (*rPFS*), which measures cancer-related fatigue on multiple dimensions (e.g., sensory, affective, cognitive, and behavioral) on 10-point scales [37]. This scale has good reliability and validity with internal consistency ranging from 0.7 to 0.9 across 4 dimensions in previous studies of patients with cancer [37,38]. Also administered was the 7-item Patient Reported Outcomes Measurement Information System-Fatigue subscale (PROMIS-F), which had an internal consistency reliability coefficient of 0.81 when validated in multiple disease populations including cancer [39]. Fatigue is essentially a subject experience and measurement of fatigue is a challenging process [40]. Utilizing two validated fatigue instruments for a biomarker-directed hypothesis strengthens our findings. Depressive symptoms were screened using the Hamilton Depression Rating Scale (HAM-D) at each time point. HAM-D is a 21-item, clinician-rated paper questionnaire with good internal reliability (α = 0.81 to 0.98) [41].

### 4.1. Gene Expression Chip Processing

At each time point, 2.5 mL of blood from each subject was collected using RNA PAXGene tubes (Qiagen, Frederick, MD, USA) and stored at −80 °C freezer until ready for RNA extraction. Total RNA were extracted from leukocytes following the PAXgene blood kit procedure (PreAnalytix, Valencia, CA, USA). RNA extraction, purification, cDNA and cRNA synthesis, amplication, hybridization, scanning, and data analyses were conducted following standard protocols as previously described [42]. Summary of Affymetrix microarray chips (HG U133 Plus 2.0, Santa Clara, CA, USA), normalization of raw signal intensity values, transformation of quantile normalization value, detection of outliers, and statistical methods used followed the same procedures that were previously described [14]. The most up-regulated gene was selected for confirmation by quantitative real time polymerase chain reaction (qPCR) and enzyme-linked immunosorbent assay (ELISA).

### 4.2. Confirmatory Quantitative Real Time Polymerase Chain Reaction (qPCR)

A total of 100 to 150 ng of extracted RNA per sample was converted to cDNA using the RT^2^ First Strand Kit (Qiagen, Frederick, MD, USA) and subsequently diluted tenfold with dH_2_O. Q-PCR amplification mixers (10 μL) contained 1 μL of diluted first strand cDNA, 5 μL of 2 × RT2 Real Time SYBR Green/Rox PCR Master Mix (Qiagen, Frederick, MD, USA) and 400 nM of forward and reverse primers. *GADPH* and *ACTB* were used as reference genes. Primers for *GAPDH* (reference position 756), ACTB (reference position 730), and *IFI27* (reference position 149) were used for normalization of data and amplification of Interferon, alpha-inducible protein 27 (Qiagen, Frederick, MD, USA). All samples were tested in triplicate. Reactions were carried out on ABI PRISM 7900HT Sequence Detection System and were subjected to initial ten minute denaturation at 95 °C and 40 cycles at 95 °C for 15 seconds and 60 °C for 60 seconds. When calculating for delta Ct values, geometric means of Ct values of the 2 reference genes were used.

### 4.3. Confirmation by Enzyme-Linked Immunosorbent Assay (ELISA)

*IFI27* expression was confirmed using the human interferon alpha-inducible protein 27, mitochondrial isoform 1 ELISA kit (Cusabio, Wuhan, China, catalog # CSB-EL011009HU). About 3 mL of whole blood collected using EDTA tubes were centrifuged (1400× *g* for 10 minutes at 4 °C) and cells were separated and stored at −80 °C in a freezer. The cell pellets (erythrocytes and white blood cells) were thawed on ice and lysed in 2 volumes of cell extraction buffer (10 mM Tris HCl, pH 7.4, 100 mM NaCl, 1 mM EDTA, 1mM EGTA, 1% Triton, 0.1% SDS and 10% glycerol with protease inhibitors). Protein concentrations were determined using Pierce^®^ BCA protein assay kit (Thermo Scientific, Rockford, IL, USA). ELISA was performed using 100 μL of diluted cell lysate samples following manufacturer’s instruction. All samples were tested in triplicate. The final concentration of each sample was normalized to the amount of cell lysate (mg). The plates were read in a micro plate reader VICTOR^3^ at 450 nm for 0.1 s.

### 4.4. Statistical Methods

Descriptive statistics were calculated for the participants’ demographic and clinical characteristics. Gene expression data were analyzed using Partek Genome Suite version 6.12 (Partek Inc., St. Louis, MO, USA). Differentially expressed genes were selected using the following criteria: fold change >2.5 or <−2.5 and a *p*-value of <0.01. We used independent *t*-tests to describe the mean differences of fatigue and gene/protein expressions from subjects at baseline (D0) and matched controls. Repeated-measures ANOVA was used to compare the mean differences of fatigue and gene/protein expressions at baseline, midpoint, and at the end of the treatment for the EBRT subjects. Individual growth curve analysis was used to describe how variables change over time during EBRT from baseline to the end of the treatment. All statistical analyses were conducted using SPSS 19.0 (IBM Corporation, Armonk, NY, USA), and R 2.15.0 for window (available online: http://cran.r-project.org/bin/windows/base/old/2.15.0/).

Linear mixed effect model was used to determine the association between changes in fatigue and gene/protein expression. The intercept and slope of the individual growth curve for fatigue scores, gene expression, and protein concentrations were estimated using mixed model analysis. In the model, we used time variables in terms of days during EBRT and a simple linear relationship was assumed in the time variable. The intercepts and slopes of the outcome variables (changes in fatigue scores) and the predictors (changes in gene expression and changes in protein concentration) for each participating individual were estimated in the mixed model. In addition to the analyses, the changes over time were modeled in a linear fashion. The slope of the individual growth curve represents the estimated rate of change over time during EBRT and the intercept of the individual growth curve represents the estimated baseline value for each patient based on a linear trajectory of their fatigue scores. To determine the associations between changes in fatigue scores, gene expression, and protein concentration, the intercepts and slopes of fatigue scores and clinical predictors (gene and protein expressions) were estimated based on a mixed model for each patient and the resulting estimated intercepts and slopes were correlated. The missing data were not assigned any replacement values and were not used for the analysis.

## 5. Conclusions

Fatigue is a commonly reported symptom and can be debilitating for many cancer patients at any stage of the disease or even during survivorship. Without knowing the molecular-genetic etiology of fatigue induced by cancer and/or its treatment, interventional options to manage it will remain challenging. Identification of possible biomarkers for fatigue associated with cancer may provide insights on possible therapeutic targets. Determining the functional significance of the association between fatigue symptoms and *IFI27* expression may identify key novel pathways, for example pro-apoptotic signals, oxidative stress, or para-inflammatory bystander response, that supports the proposed assumption that inflammation and mitochondrial dysfunction play major roles in the intensification of fatigue during cancer therapy.

## Supplementary Information



## Figures and Tables

**Table 1:** Demographic and clinical characteristics of study sample at baseline.

Variables	Participants (*n* = 40)		*p* value

	Patients (*n* = 20) Mean (±SD) or *N* (%)	Controls (*n* = 20) Mean (±SD) or *N* (%)	
**Mean Age, years**	65.6 ± 7.5	62.8 ± 6.1	0.36

**T-stage**			

T1c	7 (35%)	15 (75%)	
T2a	7 (35%)	5 (25%)	
T2b- T2c	3 (15%)		
T3a- T3b	3 (15%)		

**Gleason Score**			

6–7	10 (50%)	20 (100%)	
8–10	10 (50%)		
Karnofsky performance scale	89.5 (±2.2)	95.2 (±1.2)	0.91
Testosterone (ng/dL)	211.7 (±167.5)		
Thyroid Stimulating Hormone (μIU/mL)	2.01 (±1.1)		
PSA (ng/mL)	15.8 (±13.2)	2.68 (±1.9)	0.02
Albumin (g/dL)	3.9 (±0.3)		
Hemoglobin (mg/dL)	13.7 (±0.9)		
Hematocrit (%)	40.3 (±3.8)	42.1 (±1.7)	0.22
Depression (HAM-D)	1.2 (±2.1)	0.5 (±0.6)	0.11
Fatigue score (*rPFS*)	1.51 (±1.4)	1.46 (±1.7)	0.93

**Total EBRT dosage (Gray)**			

75.6	18 (90%)		
68.4	2 (10%)		

ng/dL = nanogram per deciliter; μIU/mL = micro International Units per milliliter; ng/mL = nanogram per milliliter; PSA = prostate specific antigen; g = gram; mg = milligram; Day 0 = baseline, before external beam radiation therapy (EBRT); HAM-D = Hamilton Depression Scale; *rPFS* = Revised Piper Fatigue Scale.
