# Supplementary material for: The Association of IFI27 Expression and Fatigue Intensification during Localized Radiation Therapy: Implication of a Para-Inflammatory Bystander Response
Source: Int J Mol Sci. 2013 Aug 16;14(8):16943–57. doi: 10.3390/ijms140816943 (PMC3759944; doi:10.3390/ijms140816943)
Supplement: Supplementary file 1 [file ijms-14-16943-s001.pdf]

Supplementary Information

Table S1. Top 10 Differentially expressed genes by microarray.

Gene symbol	Gene name	Expression value	*p* value
IFI27	**Interferon alpha-inducible protein 27**	**0.774**	$p < 0.0001$
CA1	Carbonic anhydrase 1	0.705	$p < 0.0001$
HBD	Hemoglobin subunit delta	0.640	$p < 0.0001$
XK	X-linked Kx blood group	0.534	$p < 0.0005$
RHCE/RHD	Blood group Rh(CE) polypeptide	0.507	$p < 0.0005$
MS4A1	B-lymphocyte antigen CD20	−0.821	$p < 0.0001$
IGHM	Ig mu chain C region	−0.816	$p < 0.0001$
PAX5	Paired box protein Pax-5	−0.791	$p < 0.0001$
FCRLA	Fc receptor-like A	−0.669	$p < 0.0001$
TTC3	Tetratricopeptide repeat protein 3	−0.647	$p < 0.0005$

© 2013 by the authors; licensee MDPI, Basel, Switzerland. This article is an open access article distributed under the terms and conditions of the Creative Commons Attribution license (http://creativecommons.org/licenses/by/3.0/).
